# Spasticity in multiple sclerosis and role of glatiramer acetate treatment

**DOI:** 10.1002/brb3.367

**Published:** 2015-07-14

**Authors:** Jose Eustasio Meca-Lallana, Rocío Hernández-Clares, Ester Carreón-Guarnizo

**Affiliations:** 1 Department of Neurology, Multiple Sclerosis Unit, Hospital Clínico Universitario Virgen de la ArrixacaCarretera Madrid-Cartagena s/n, 30120, Murcia, Spain; 2 Cátedra de Neuroinmunología Clínica y Esclerosis Múltiple, UCAM Universidad Católica San Antonio de MurciaCampus de los Jerónimos, Guadalupe, 30107, Murcia, Spain

**Keywords:** Clinical effectiveness, glatiramer acetate, multiple sclerosis, muscle spasticity

## Abstract

**Introduction:**

Spasticity is one of the most disabling and difficult-to-treat symptoms shown by patients with multiple sclerosis, who often show a suboptimal and unsatisfactory response to classic treatment and new available nonpharmacological alternatives. Due to the progressive nature of this condition, the early management should be essential to improve long-term outcomes.

**Methods:**

We performed a narrative literature review of the contribution of spasticity to the burden of multiple sclerosis and the potential role of classic disease-modifying drugs.

**Results:**

Added to the underlying pathophysiology of spasticity, certain external factors and drugs such as interferon may exacerbate the existing condition, hence their awareness is crucial as part of an effective management of spasticity. Furthermore, the evidence for the effectiveness of glatiramer acetate in preventing spasticity in naïve patients and in those switching from interferon should not be ignored.

**Conclusions:**

This literature review proposes the examination of spasticity and the influence of classic disease-modifying agents on the level of existing condition among the variables to be considered when deciding on therapy for multiple sclerosis in clinical practice.

## Introduction

Spasticity is one of the most prevalent symptoms in multiple sclerosis (MS) (Barnes et al. 2003; Rizzo et al. 2004; Berger 2013; Collongues and Vermersch 2013; Oreja-Guevara et al. 2013a), and the one most affecting the quality of life (QoL) and functionality of patients (Hemmett et al. 2004; Fernandez et al. 2011; Arroyo et al. 2013; Zettl et al. 2014), but its pathophysiology is complex and not fully understood as noted in various review articles (Pappalardo et al. 2006; Kheder and Nair 2012; Amatya et al. 2013; Oreja-Guevara et al. 2013b). Classically characterized by a velocity-dependant intrinsic resistance to passive movement of a limb in people with upper motor neurone syndrome (Lance 1980) was redefined by the EU-SPASM as “a disorder of sensory motor control caused by an upper motor neuron lesion that manifests as intermittent or sustained activation of muscles” (Stevenson 2010). This definition considers the role of viscoelastic properties of soft tissue to limb stiffness and proprioceptive and cutaneous neural pathways.

Epidemiological studies already indicated that spasticity may affect up to 80% of patients with MS (Rizzo et al. 2004); and that was an important factor contributing to disability in this population (Beard et al. 2003). Recent several survey-based studies have updated the information on the prevalence of this condition. The study by the Multiple Sclerosis International Foundation was an online survey of 692 patients with MS showed that 75% of the survey respondents experienced spasticity in both legs accompanied by main symptoms of stiffness or tension in 88.7% of cases (Spasticity Online Survey Results, 2008). In the more recent Spanish study “6E” (Oreja-Guevara et al. 2013a), 65.7% of the more 2000 patients with MS had spasticity, and 40% described it as moderate to severe; some limitations of this study was the true response rate of only 30% and the analysis of reported spasticity based on disease duration instead of the level of disability.

Retrospective studies in Spain (Arroyo et al. 2011) and Germany (Henze et al. 2013) confirm the frequent occurrence of spasticity in patients with MS (Berger 2013). A survey on epidemiology and management of MS worldwide and across the European Union (EU), aimed at 157 specialized healthcare professionals (95% neurologists), reported similarity between the EU and the rest of word respondents in the epidemiology of MS. The results of approximately 40% of patients with spasticity, being moderate or severe in 35% and 25% of cases, respectively, highlight unmet needs related to its clinical management (Collongues and Vermersch 2013).

## Clinical and Economic Impact of Spasticity in MS

Spasticity presents as an increase in muscle stiffness, often accompanied by spasms and altered reflexes; the natural course of the disease may end up leading to highly disabling conditions with muscle retraction and osteoarticular deformities, so early therapeutic intervention is essential (Vivancos-Matellano et al. 2007).

In cases such as those with a high degree of weakness in lower extremities, a certain level of spasticity may even be positive for the patient, in the sense that the patient makes use of the stiffness or spasms to aid in rising or moving (Pappalardo et al. 2006). Nevertheless, the constant severe stiffness or frequent spasms that accompany progression of the disease have a decisive impact on the patient’s functional autonomy, and may cause a high level of immobility (Beard et al. 2003). This interferes with the patient’s personal hygiene, gait, manipulation and transfer of objects, and limits his/her relationship with work, social, and the family environment. The complications for the patients may go further in terms of discomfort or pain, altered sleep and sexual activity, bladder dysfunction, emotional disturbances, anxiety, and depression. Furthermore, worsening of these associated symptoms is positively correlated with an increased degree of spasticity (Oreja-Guevara et al. 2013a). In particular, the percentage of patients experiencing daily spasms, sleep alterations, and urinary dysfunction increases as spasticity worsens (Oreja-Guevara et al. 2013a), as well as mean symptom scores (Zettl et al. 2014).

In short, spasticity is a debilitating symptom of MS that is directly correlated with the progression of disability (Shakespeare et al. 2003), and with the number of relapses in the last 12 months (Oreja-Guevara et al. 2013a). It also interferes with the performance of daily activities (Barnes et al. 2003; Hemmett et al. 2004; Zwibel 2009; de Sa et al. 2011; Oreja-Guevara 2012) and is directly associated with a reduction in QoL, particularly of the physical component (Rizzo et al. 2004; Wu et al. 2007; Arroyo et al. 2013).

Spasticity may affect activities of daily living in up to 44% of patients with MS, and may even prevent their development in 4% (Rizzo et al. 2004). This percentage may increase from 10% in cases of mild spasticity to 85% in those with severe spasticity (Flachenecker et al. 2014). Daily life is mainly affected by the limitations related to mobility (Donze and De Sèze 2012), which for 66% of patients are one of the most worrying symptoms of spasticity together with stiffness for 74% (Flachenecker et al. 2014).

Few studies have been conducted to evaluate the impact of spasticity on QoL. Recent data have shown a reduction in the mean score of the EQ-5D from 0.6 to 0.3 with increased severity of spasticity (Flachenecker et al. 2014), and a significant correlation between scores on the SF-12 questionnaire and those obtained on the Ashworth and Numerical Rating Scale (NRS) spasticity scales (Arroyo et al. 2013).

Patients with MS also suffer impairment of their work productivity, with spasticity being one of the associated symptoms that may contribute most to significantly reducing functional independence (Barnes et al. 2003). In the study of Rizzo et al. (2004), over 50% of patients with mild, moderate, or severe spasticity were unemployed, a significantly higher percentage than in patients without spasticity (28.7%). In turn, Oreja-Guevara et al. (2013a) reported a significantly higher rate of retired patients in those who had spasticity (34.7% vs. 8.6%), and contrarily, a significantly lower rate of active patients (43.4% vs. 64.5%). Indeed, results pointing to that worsening of spasticity reduces the ability to perform an active job have been found (Oreja-Guevara et  al. 2013a). Despite the socioeconomic consequences of MS is not only attributable to spasticity, this has a substantial economic impact on the society not only in terms of loss of productivity as noted, but because of drug costs and healthcare resources utilization (HRU), which is also directed correlated to the degree of spasticity. (Oreja-Guevara et al. 2013a). The impact that greater severity of spasticity has on HRU (Tyry et al. 2013; Zettl et al. 2014) may triple the cost derived from management of mild spasticity (Zettl et al. 2014). These findings support the importance of an early intervention to minimize the impact of spasticity not only on MS patients’ quality of life but on the social and health-related costs. Nonetheless, data on economic burden of spasticity associated with MS are scarce, and limited to national studies with small sample sizes. Indirectly, larger studies that have evaluated the impact of MS on the socioeconomic burden have helped to describe the contribution of these symptoms of MS to the burden of the disease. The mean annual cost of a patient with MS and spasticity varies between countries, in Sweden being 10 times higher (Svensson et al. 2014) than that estimated in Spain (Arroyo et al. 2011). A fact that may be due to differences in the populations studied, or to the economic weight of the resources most characteristic or representative of each country.

## Triggering and Aggravating Factors

In addition to the intrinsic physiological characteristics of spasticity, several external factors may exacerbate the existing condition, and therefore, their evaluation is important for effective spasticity management. A systematic review on the impact of physiological and psychological triggers on spasticity, which mostly included studies performed in the spinal cord injury population, did not provide conclusive data on the true effect of certain factors in spasticity (Phadke et al. 2013); however, it found objective clinical evidence of the effect of pregnancy, posture, cold, circadian rhythm, and skin conditions. Aspects related to the menstrual cycle, bowel and bladder problems, and stress, appear to subjectively aggravate spasticity. If these findings could be similar to other neurological diseases remains unclear. In fact, the role of triggers on spasticity in MS is virtually unknown, and probably the referred extrinsic factors do not constitute a common cause. The main triggering or aggravating factors of spasticity described in MS include urinary tract or other infections, excessive fatigue, stress, pressure sores, pain, constipation, fever, and environmental temperature extremes, as well as some medicines such as antidepressant drugs and immunomodulatory agents such as interferon (Kheder and Nair 2012; Phadke et al. 2013).

The evidence on antidepressant treatment and spasticity is scant, old, and limited to a few clinical case reports. Briefly, there are studies indicating that selective serotonin reuptake inhibitors exacerbate spasticity (del Real et al. 1996; Stolp-Smith and Wainberg 1999), possibly due to the effects of serotonin on the motor neuron and reflex activity (Stolp-Smith and Wainberg 1999); more recently, a review included spasticity among the characteristic symptoms of serotonin syndrome (Talarico et al. 2011). Although in another line, there are published cases series reporting increased spasticity after systemic naloxone infusion in patients with spinal cord injury (Brackett et al. 2007), indicating a relationship between opioid neuromodulation and spasticity. A case of spasticity induced by lamotrigine toxicity has been also documented (Algahtani et al. 2014). Given the possibility that some of these triggers result in a self-perceived increase in spasticity, it is proposed that they be identified using patient perception and objective measures of spasticity (Phadke et al. 2013).

## Clinical Evaluation of Spasticity

Clinical assessment of spasticity is based on evaluation of the range of motion of the upper and lower extremities and its impact on patient functionality. As described by Katz and Rymer (1989), spasticity is an entity more difficult to characterize than to recognize, and even more difficult to quantify, among other reasons because of the discrepancy between the patient’s subjective judgment and clinical measurement (Gomez-Soriano et al. 2012). Neurophysiological studies, biomechanical techniques, and clinical scales can be used despite that the former two are questioned due to the low correlation with clinical indicators of spasticity, and problems of reliability and sensitivity (Voerman et al. 2005; Wood et al. 2005). Clinical scales, however, are useful in clinical practice (de Sa et al. 2011) although there is no consensus on the most appropriate. The heterogeneity of clinical expression leads to recommend the combined application of various scales to quantify the different signs of spasticity (Gomez-Soriano et al. 2012), together with the evaluation of pain and other related symptoms to obtain a comprehensive clinical evaluation (Rekand 2010; Pozzilli 2013).

The Ashworth scales (Ashworth 1964; Bohannon and Smith 1987) are currently the most used in clinical practice despite the fact that their validity and reliability are questioned (Ansari et al. 2006, 2008; Fleuren et al. 2010); a new modified version of the Ashworth scale (Ansari et al. 2008) is providing encouraging results (Ansari et al. 2008, 2009; Ghotbi et al. 2009, 2011). Other widely used scales are the parametric NRS 0-10 spasticity scale (Farrar et al. 2008), which assesses in a reliable and valid manner, even superior to the previous Ashworth scale, the intensity of spasticity perceived by the patient, and the PSFS (Penn Spasm Frequency Scale) for quantifying the number of spasms in the affected limb during the day.

The development of the MSSS-8 scale (Hobart et al. 2006) offered a new perspective in clinical evaluation of spasticity by considering the patients’ experience and perception of the impact of spasticity on their daily life, which coincides with alterations of the spatio-temporal parameters of gait (Balantrapu et al. 2012). Additionally, a new measure of disability and functionality related to spasticity (Rekand disability and spasticity score) is being validated (Rekand 2010). The marked impact of spasticity on the QoL and general well-being of patients with MS suggest it should be evaluated either generically by the EQ-5D, SF-36, and SF-12, or specifically by the MusiQoL. Moreover, the subjective perception of the effects of spasticity in both physical and psychological domains has been found to be significantly correlated with scores on the Ashworth and NRS scales (Arroyo et al. 2013).

Despite the variability in the tools for evaluating spasticity, there is no consistency in the literature on the use of validated measures which, according to the experts, may have contributed to the inconclusive results on the efficacy of antispastic agents currently in use obtained from placebo-controlled studies and comparative studies (Shakespeare et al. 2003).

## Symptomatic Treatment of Spasticity

With increased life expectancy of patients with MS, management of associated symptoms is gaining increasing importance (Hartung 2012). In this regard, numerous efforts are being focused on management of spasticity not only because it is the main cause of disability in MS, but because it shows a limited response to traditional treatment (Shakespeare et al. 2003; Oreja-Guevara 2012), which in many cases is suboptimal (Barnes et al. 2003), and unsatisfactory for 40% (Collongues and Vermersch 2013; Flachenecker et al. 2014) or 55% of health care professionals (Henze et al. 2013) in terms of efficacy and safety. A large Spanish survey showed a high percentage of patients with moderate (58%) or severe spasticity (47%) untreated (Oreja-Guevara et al. 2013a), compared to 31% and 21%, respectively, in a similar study from the United States (Rizzo et al. 2004), and 16% in a German registry for cases of severe spasticity (Henze et al. 2013; Flachenecker et al. 2014). Other studies have reported that approximately 50% of patients with spasticity receiving treatment require a dose adjustment or use of additional treatment (Barnes et al. 2003; Arroyo et al. 2011).

Pharmacological agents and/or nonpharmacological interventions are the mainstay of treatment of spasticity (Stevenson 2010), with surgical treatment being recommended in carefully selected cases refractory to other management strategies (Oreja-Guevara et al. 2013b). Oral antispastic agents such as baclofen, diazepam, dantrolene, and tizanidine are widely used to improve spasticity, though they shown limited effectiveness and a safety profile that may limit their usefulness (Beard et al. 2003; Shakespeare et al. 2003; Stevenson 2010). A Cochrane systematic review concluded that these drugs offered little overall benefit and that evidence regarding efficacy and tolerability was poorly documented. The cannabis extract nabiximols is restricted to patients refractory to conventional treatment, and invasive treatments such as local injection of botulinum toxin (BoNT), or intrathecal baclofen infusion to cases with greater disability (Paisley et al. 2002; Oreja-Guevara et al. 2013b).

The nonpharmacological interventions and complementary and alternative measures for treating spasticity has been shown in a Cochrane systematic review in 2012 (Amatya et al. 2013), and in a more recent summary of evidence-based guidelines (Yadav et al. 2014) as having a low level of evidence for effectiveness, which limits their clinical use.

Briefly, exercise improves spasticity in MS (Tarakci et al. 2013) and combined with intermittent transcranial magnetic theta burst stimulation (iTBS) reduces the spasticity measured with the Modified Asworth Scale (MAS) and MSSS-88 scales (Mori et al. 2011),

Physiotherapy may be more effective than physical exercise, and even provide an advantage derived from combined use (Negahban et al. 2013). Furthermore, as additional therapy to treatment with BoNT, physiotherapy increases the reduction in spasticity even after 12 weeks, and may improve the overall response to treatment (Giovannelli et al. 2007). The evidence of a certain benefit of reflexology in the treatment of spasticity is limited to a small study with 70 patients that showed a significant improvement in the mean score on MAS after 11 weeks of treatment compared to the control group that had received a nonspecific massage in the shin area (Siev-Ner et al. 2003). (Schyns et al. 2009). There is also no evidence that performing other physical activities such as yoga or sports climbing has any effect on spasticity (Velikonja et al. 2010).

Effective management of spasticity should be aimed at preventing or minimizing the triggering or aggravating factors, as well as reducing spasticity and preventing its consequences (Ward 2002; Henze et al. 2006; Rekand 2010), all coordinated by a multidisciplinary team involving the family and caregivers, and which help the patient to manage their condition through education and access to treatment strategies (Stevenson 2010). Vivancos-Matellano et al. (2007) developed a guide to comprehensive treatment of spasticity in MS that provides a rational and global approach to this disease. These education and management strategies of spasticity should be implemented early to prevent or reduce its severe complications, since it is a chronic and changing symptom that progresses to a higher degree severity as the disease advances (Pappalardo et al. 2006).

Based on the available scientific evidence, a group of Spanish specialists in MS from the Demyelinating Diseases Group of the Spanish Neurology Society has prepared a consensus document to establish uniform criteria for treatment of spasticity, which facilitates therapeutic decision making in routine clinical practice (Oreja-Guevara et al. 2013b). The European guidelines for the management of spasticity in MS are currently being developed, and coordinated by the European Federation of Neurological Societies (Gold and Oreja-Guevara 2013).

## Potential Role of Disease-modifying Drugs on Spasticity in MS

Interferon beta and glatiramer acetate (GA) are effective in RRMS and CIS patients. However, their impact on symptoms such as spasticity and fatigue are not usually considered in clinical practice despite being as important as relapses (Miller et al. 2011) and disability (Goksel et al. 2011) in determining the QoL in patients with MS. Nor a recommendation for or sequence of DMDs based on the presence or the prevention of these symptoms is established, probably because the benefits of these drugs on symptoms and impairments have not been clearly established (Zwibel 2009).

## Interferon Beta

Though limited, there is evidence of increased spasticity during interferon therapy, a phenomenon which, according to some authors (Leary and Thompson 2004), is not surprising given that worsening of existing spasticity associated with intercurrent symptoms such as fever is common in MS. However, in the opinion of other authors, it is a fact that should be taken into account when deciding on interferon therapy (Frese et al. 1999).

Among the first observations in this regard is the study by Lublin et al. (1996), a review on the management of patients with RRMS treated with interferon beta-1b based on their experience with the drug and as principal investigators of the pivotal trial for approval of the drug. This study, together with the phase III study of interferon beta-1b in SP forms of the disease (The IFNB Multiple Sclerosis Study Group 1993), showed a transient increase in spasticity after interferon beta-1b therapy in 13% and 37.8% of patients, respectively. It was a symptom especially sensitive to worsening with interferon beta therapy (Walther and Hohlfeld 1999), whose management rarely required a temporary reduction in the dose of the drug (Lublin et al. 1996; Munschauer and Kinkel 1997).

The open-label study conducted by Bramanti et al. (1998) in primary progressive (PP) forms also showed a frequent and clinically relevant increase in spasticity experienced by patients treated with interferon beta-1b, after a mean of 2 months of treatment, which improved some months after discontinuation of interferon. Specifically, from a total of 19 patients treated with interferon beta-1b, 13 (68%) manifested a significant increase in spasticity, and 7 (37%) decided to discontinue interferon beta-1b therapy 6 months after starting the treatment. According to the authors, this increase in spasticity was not correlated with any new pathology on magnetic resonance imaging, and probably was due to a direct effect of the drug on motor neurons, or a consequence of previous central control.

Frese et al. (1999) retrospectively analyzed the data of 90 patients with RRMS treated with interferon beta-1b, with the aim of determining the reasons for treatment discontinuation. The results showed that severe spasticity was the most important cause of discontinuation of interferon beta-1b in patients with long-duration disease (mean 10.4 ± 3.3 years) and high disability (mean EDSS 5.0 ± 1.1). The occurrence of spasticity attributed to interferon beta-1b appeared some hours after injection and lasted at least for 2 days.

Also in PP forms, Leary and Thompson (2004) conducted a clinical trial in 50 patients randomized to receive weekly for 2 years interferon beta-1a 30 *μ*g (15 patients), interferon beta-1a 60 *μ*g (15 patients), or placebo (20 patients). Contrary to what was reported in the mentioned studies, this study did not show a significant increase in spasticity in patients with PPMS treated with interferon beta-1a, though a trend can be noted toward increased spasticity in both treatment groups (33%) versus placebo (15%). In addition, a sustained increase in spasticity was less frequent in the interferon beta-1a 60 *μ*g (13%) compared to interferon beta-1a 30 *μ*g (60%) group.

An open-label 2-year follow-up study (Flechter et al. 2002) comparing GA 20 mg sc (daily or alternate day administration) and interferon beta-1b (8 MIU) in 58 patients with RRMS showed increased spasticity of lower limbs only in the interferon beta-1b group, with a rate of 15%. It should be noted that both groups were comparable in terms of sex, age, disease duration, number of relapses in the 2 years prior to the start of treatment, and degree of disability (EDSS).

## Glatiramer Acetate

Glatiramer acetate (GA) has been shown to have a clear beneficial effect on spasticity in patients with RRMS previously treated with interferon beta. In treatment-naïve patients, its effect has been shown to be less pronounced, but without evidence of worsening of spasticity. Only two studies have been conducted to evaluate this effect, which is summarized below, and whose main data are given in Table 1993. The first of the two studies, published in 2010 by Meca-Lallana et al. was a prospective observational pilot study conducted in two cohorts of patients with RRMS and spasticity who were going to be treated with GA: a cohort of patients previously treated with interferon beta who switched treatment for reasons of safety or lack of efficacy (*n* = 13) and a cohort of naïve patients (*n* = 15). After 18 months of follow-up and compared to baseline, all patients who switched from interferon beta to GA showed a significant reduction in mean scores on the MAS for the right hemibody (1.18 [0.60] vs. 1.85 [0.61]; *P* = 0.002) and left hemibody (1.27 [0.65] vs. 1.86 [0.55]; *P* = 0.045), and also in scores of the PSFS (0.36 [0.81] vs. 2.00 [0.91]; *P* = 0.002) and Global Pain Scale (GPS) (24.09 [17.15] vs. 47.69 [13.94]; *P* = 0.002). In patients who started GA as the first disease-modifying drug, no change was seen in their degree of spasticity on any of the clinical scales used, probably because they had a baseline mean degree of spasticity that was very mild and lower than those treated with interferon beta. However, they did show a significant reduction in H-reflex latency in the left hemibody (28.75 [2.01] vs. 30.31 [2.44] at baseline; *P* = 0.005), and H/M ratio in the right hemibody (0.35 [0.19] vs. 0.45 [0.15]; *P* = 0.025), two objective electrophysiological indicators of improvement in spasticity, whose sensitivity, validity and reproducibility have been previously confirmed (Levin and Hui-Chan 1993; Pisano et al. 2000; Joodaki et al. 2001; Pizzi et al. 2005), and as such can give consistency to other assessment measures of spasticity with methodological limitations (Bakheit et al. 2003). Nevertheless, few studies have evaluated response on neurophysiological tests in combination with clinical assessment measures, including this study. Furthermore, the results found are inconclusive, showing from a good relationship between electrophysiological parameters and the scores obtained on the MAS (Skold et al. 1998; Sorinola et al. 2009) to a poor to moderate relationship (Blackburn et al. 2002) or no relationship (Heidari et al. 2011).

**Table 1:** Efficacy of glatiramer acetate (GA) in the spasticity of relapsing-remitting multiple sclerosis (RRMS). Results of the only two prospective studies currently available

Study	Patients	Assessment points	Summary of the results	Favorable outcomes to GA	Author’s conclusions
Meca-Lallana (2010)	Cohort 1: Patients with RRMS and spasticity who were being switched to GA from interferon beta (*n* = 13)	Cohort 1: Baseline, and 18 months	Cohort 1: –Significant improvement in MAS, PSFS and GPS scores.	Cohort 1: –MAS for the right hemibody (from 1.85 to 1.18; *P* = 0.002).	AG significantly improves spasticity among patients previously treated with interferon beta.
		Cohort 2: Baseline and 12 months	–No significant differences for ATRS, EDSS, H-reflex latency, or amplitude on either side, or lower limb H/M ratio on either side	–H/M ratio on the right side (from 0.45 to 0.35; *P* = 0.025) –PSFS (from 2.00 to 0.36; *P* = 0.002). –GPS (from 47.96 to 24.09; *P* = 0.002).	Although the effect was less pronounced in naïve patients, the spasticity was not worsened
	*Cohort 2:* Patients with RRMS and spasticity who were treatment naïve (*n* = 15)		Cohort 2: –Significant improvement in H-reflex latency of the left side and H/M ratio on the right side.	Cohort 2: –H-reflex latency on the left side (from 30.31 to 28.75: *P* = 0.005).	
			–No significant improvements in MAS, PSFS, GPS, ATRS, EDSS, H-reflex latency on the right side, H-reflex amplitude on either side, or lower limb H/M ratio on the left side	–MAS for the left hemibody (from 1.86 to 1.27 *P* = 0.045).	
Meca-Lallana (2012)-Escala study	Patients with RRMS, EDSS ≤ 5, and spasticity who have switched from interferon beta to GA for at least 24 weeks (*n* = 68)	Baseline, and months 3 and 6	–Significant improvements in clinical spasticity (PSFS, MAS, highest MAS, ATRS, GPS) at 3 and 6 months.	–PSFS (from 1.7 to 1.4 at 3 months; *P* < 0.01; and to 1.3 at 6 months; *P* < 0.01).	Switching from interferon beta to GA improves spasticity in terms of spasm frequency, muscle tone and pain even at 6 months of treatment
			–Significant decrease in patients without spasmolytic treatment compared to those receiving it in GPS and MAS scores at baseline, and months 3 and 6.	–MAS (from to 0.7 to 0.6 at 3 months; *P* < 0.01, and to 0.5 at 6 months; *P* < 0.01).	Beneficial effect of GA on quality of life, and absenteeism from spasticity.
			–In these patients, significant decrease in highest MAS scores at moths 3 and 6, and PSFS and ATRS at month 6.	–Highest MAS (from 1.9 to 1.7 at month 3; *P* < 0.01, and to 1.5; *P* < 0.01).	
			–Significantly improvement in QoL at 6 months from baseline in all items except sexual function, and overall QoL means.	–ATRS (from 1.6 to 1.4 at 3 months; *P* < 0.01, and to 1.3 at 6 months; *P* < 0.01).	
			–Any working day lost due to spasticity-related symptoms	–GPS (from 29.4 to 24.7 at 3 months; *P* < 0.01, and to 22.1 at 6 months; *P* < 0.01).	
				–Improvements in patients without spasmolytic treatment but not in those receiving it:	
				Baseline:–GPS (26.3 vs. 34.7; *P* < 0.05). –MAS (0.6 vs. 0.9; *P* < 0.01). Month 3: –GPS (20.3 vs. 32.1; *P* < 0.05).	
				–MAS (0.4 vs. 0.8; *P* < 0.01). –Highest MAS (1.5 vs. 2.0; *P* < 0.05)	
				Month 6: –GPS (15.0 vs. 26.1; *P* < 0.01). –MAS (0.4 vs. 0.8; *P* < 0.01). –Highest MAS (1.2 vs. 2.0; *P* < 0.01) –PSFS (1.1 vs. 1.5; *P* < 0.01). –ATRS (1.2 vs. 1.7; *P* < 0.01)	

MAS, Modified Ashworth Scale; PSFS, Penn Spasm Frequency Scale; GPS, Global Pain Scale; ATRS, Adductor Tone Rating Scale; EDSS, Expanded Disability Status Scale; QoL, Quality of life.

The results of this first study with GA were supported by a subsequent multicenter study (the ESCALA study) in patients with RRMS and confirmed spasticity who switched to GA after interferon beta (Meca-Lallana et al. 2012). Three to six months after starting GA treatment, a significant reduction was observed in scores on all spasticity assessments used, including the PSFS, MAS, Adductor Tone Rating Scale, and GPS (Table 1993). Patients experienced an improvement in spasticity from the first months of treatment with GA, regardless of the baseline degree of spasticity, resulting in improved QoL and work activity of the patient. It is true that no association between spasticity and quality and work absenteeism can be derived from this study as the correlation between variables was not analyzed, but it suggests an indirect effect of spasticity on QoL and productivity through its impact on physical and mental status, as shown by other studies (Rizzo et al. 2004; Wu et al. 2007). In fact, starting treatment with GA reduces the days of work absenteeism for reasons of disability (Rizzo et al. 2004; Lage et al. 2006; Ziemssen et al. 2008), and may be a consequence of the improvement seen in spasticity, given that the number of patients reporting absenteeism due to disability was decreased (Meca-Lallana et al. 2012).

The results of a subanalysis of the ESCALA study (Meca-Lallana et al. 2012) in patients receiving or not spasmolytic treatment showed significant improvements in all measures of spasticity used in patients without baseline spasmolytic treatment (Table 1993). According to the authors, these findings suggest that the reduction observed in spasticity was associated with GA treatment and independent of spasmolytic treatment. Although the underlying mechanism of the reduction in spasticity associated with GA has not been defined, nor to what extent the effect of GA treatment may or may not be related to its effect on disease activity, these findings led us to the notion that spasticity would be considered among factors that influence the choice of immunomodulatory therapy in patients with MS (Table 2014), and even the decision concerning if or when to switch between these two disease-modifying agents.

**Table 2:** Spasticity as clinical determinant influencing the initial treatment choice for patients with relapsing-remitting multiple sclerosis

Clinical or radiological MS activity (Hartung et al. 2011; Yamout et al. 2013; Wingerchuk and Carter 2014)
• Recent attack frequency, severity and recovery
• Lesion burden and presence of active enhancing lesions evident on brain and spinal cord MRI
• Degree of neurological impairment/residual neurological deficits
Drug availability and cost (Wingerchuk and Carter 2014)
Concomitant medical illnesses and medications (Wingerchuk and Carter 2014)
Concurrent symptomatic issues (Fox et al. 2006)
• Spasticity
• Fatigue
• Depression
• Headache
Agents’ tolerability profile (Hartung et al. 2011; Wingerchuk and Carter 2014)
Patient’s individual needs and preferences (Hartung et al. 2011; Heesen et al. 2013; Yamout et al. 2013; Wingerchuk and Carter 2014)
• Given the safety and dosing profiles of the individual therapies (level of evidence A)
• Patient autonomy
• Female patient’s plans to be pregnant
• Desire to avoid self-injections
• Desire to avoid specific adverse effects
Monitoring requirements (Wingerchuk and Carter 2014)

MS, multiple sclerosis, MRI, magnetic resonance imaging.

## Conclusions

The management of spasticity in multiple sclerosis is complex and, therefore, constitutes a challenge for neurologists. Besides being the symptom most affecting the QoL and functionality of the patients, it shows a limited response to traditional treatment and nonpharmacological alternatives.

If the progression of spasticity that accompanies the natural course of the MS may cause a high level of immobility, then early intervention on its clinical course should be essential to improve prognosis and associated complications.

As part of an effective spasticity management, the awareness or even the elimination of externally exacerbating factors is considered as crucial. In this sense, the evidence, although limited and well recognized, of worsening spasticity after interferon should be not ignored, nor the effectiveness GA shows in preventing spasticity. This review does not advocate for the use of a concrete drug, but encourages neurologists to consider both the evaluation of spasticity and how classic DMDs can influence the level of the existing condition when deciding on therapy for MS in clinical practice.
